# Prevalence and risk factors of HIV in Faisalabad, Pakistan –A retrospective study

**DOI:** 10.12669/pjms.301.4176

**Published:** 2014

**Authors:** Muhammad Arif Maan, Fatma Hussain, Muhammad Jamil

**Affiliations:** 1 Muhammad Arif Maan, MBBS, FCPS, D-Dermat, Department of Dermatology, Punjab Medical College/Sexually Transmitted Infections (STIs) Clinic, District Headquarter (DHQ) hospital, Faisalabad, Pakistan.; 2 Fatma Hussain, PhD, Clinico-Medical Biochemistry Laboratory, Department of Chemistry and Biochemistry, University of Agriculture, Faisalabad, Pakistan.; 3 Muhammad Jamil, MBBS, DCP, Clinical Pathology Laboratory, District Headquarter Hospital, Faisalabad, Pakistan.

**Keywords:** Epidemic, HIV, Risk factors, Sexually transmitted diseases

## Abstract

***Background & Objective: ***Although the magnitude of HIV in Pakistan has been well documented, but no record of HIV prevalence in Faisalabad region exists. A retrospective study was carried out at Sexually Transmitted Infections (STIs) clinic, District Headquarter (DHQ) hospital, Faisalabad, Pakistan to find out the prevalence of HIV and related risk factors.

***Methods: ***Between March, 2010 and December, 2012, a total of 31040 subjects were either interviewed or their medical records were reviewed. From those recruited by convenient sampling method, written informed consent was obtained and informed about the study protocol. Blood serum was tested for antibodies to HIV-1 and HIV-2 (Enzyme-linked immunosorbent assay, Western Blot).

***Results: ***On the whole, Anti-HIV was demonstrated in 173 (0.557%) of the respondents. This gives an overall HIV prevalence of 557 per 100,000.Averaged age of the patients was 49.5 years (range: 30-45) with 85.55% male. Majority of the patients were urban dwellers (87.28%), divorced or widowed (46.82%) and uneducated (50.28%). A large proportion (78%) of the patients was injection drug users. Compared to blood donation/transfusion and sexual interactions, injection drug use was the major potential risk factor for HIV infection.

***Conclusion: ***Most important finding was higher HIV prevalence in Faisalabad region as compared to the previous assessments at the national level. This reflects an alarming situation necessitating contextual preventive interventions. Precarious practices such as injection drug abuse, blood donation/transfusion needs to be amended and extramarital sexual contacts should be avoided.

## INTRODUCTION

HIV (human immunodeficiency virus) is characterized by a gradual deprivation of the human immune system, a condition better known as the acquired immunodeficiency syndrome (AIDS).HIV causes a slow but progressive death of CD4 T lymphocytes that coordinate the humoral and cellular responses in immune system. This epidemic has resulted in 25 million deaths worldwide.^1^ In Pakistan, HIV prevalence is only 0.04% in general population.^2^ A survey in 2005 described threat of an expanded HIV outbreak in Pakistan.^3^ A previous study showed the national HIV prevalence as 0.064%.^4^

Although, Pakistan currently portrays a low HIV prevalence, however, the concerns of a widespread HIV epidemic are primarily due to unsafe high-risk practices. More Pakistani men are infected with HIV than women and modes of HIV transmission include sexual relations, infectious blood and drug addiction.^2^^,^^3^^,^^5^^,^^6^ Another factor that poses serious intimidation to Pakistani population is the limited knowledge about HIV/AIDS. In view of the current scenario, a comprehensive study to characterize HIV in the local populations **is** suggested.^1^^,^^7^ A common misconception in Pakistani society is that being Muslim, HIV cannot be contracted. Such foresight demands continuous monitoring and preventive intrusions to control the spread of HIV.^2^

There are no reports on HIV prevalence among residents of Faisalabad, Pakistan. We aimed to determine prevalence of HIV and related risk factors in Faisalabad, Pakistan.

## METHODS

***Population and Sampling: ***This retrospective report covered about two and a half year period from March, 2010 to December, 2012 and included 31040 individuals visiting Sexually Transmitted Infections (STIs) clinic, DHQ hospital, Faisalabad, Pakistan. Participants were either recruited or their medical records were reviewed. From those recruited by convenient sampling method, written informed consent was obtained and they were informed about the protocol.

***Questionnaire: ***The following data were collected for each patient: age, gender, residential and marital status, occupation, education, history of sexually transmitted diseases (STD) and high risk behaviours (blood donation, blood transfusion, intravenous drugs abuse and sexual behaviours). Trained investigators pre-tested the validity of the form and some queries regarding sexual behaviours were modified. Sexual orientation (homosexual, heterosexual, bisexual) was determined. The study was anonymous.

***Biological Tests: ***From Clinical Pathology Laboratory, District Headquarter hospital, Faisalabad, Pakistan, patients’ records were procured. For new enrolments, blood samples were centrifuged and the sera were tested for the presence of antibodies to HIV-1 and HIV-2 (Enzyme-linked immunosorbent assay and Western blot analysis; Abbot and Cambridge Biotech, respectively).

***Statistical Analysis: ***All data were expressed as number (n) or mean (standard error).Potential risk factors were assessed by multivariate analysis of variance (MANOVA). The p value of less than 0.05 was considered to be significant. Statistical analysis was performed by Statistical Package for the Social Sciences (SPSS Inc. Chicago, IL, USA) software (version 15.0).

## RESULTS

Patients (n=31040) were either examined or their medical records were reviewed for HIV-1 and HIV-2.Data pertaining to demographic characteristics and risk factors is summarized in Tables I and II. Of these recruited sample, 170 and 3 tested HIV-1 and HIV-2 positive respectively. HIV-1 virus is the principal microbe while, HIV-2 is minor category. Whenever, HIV is mentioned without specifying the type of virus, it is assumed to be HIV-1. HIV-2 (0.009%) was not as prevalent as HIV-1 (0.54%).On the whole, Anti-HIV was demonstrated in 173 (0.557%) of the respondents. This gives an overall sero prevalence of 557 per 100,000.

Average age of the HIV infected population was about 50 years and majority (85.55%) were male. The age group for HIV incidence was 30-45 years, as it accounted for the 51% of the infected sample.HIV positivity was more prominent (p=.001) in male as compared to the female sample (6:1 ratio).

**Table-I:** Demographic characteristics of the patients

*Characteristics *	*Number (n)*	*P value*
HIV-1 positive	170	-
HIV-2 positive	3	-
**Age**, mean (SE), years Age range (min.-max), years	49.5 (2.73) 30-45	-
**Gender **		
Male	148	0.001
Female	25	
**Residence**		
Urban	151	0.035
Rural	22	
**Current marital status**		
Unmarried	63	0.065
Married	29	0.178
Divorced/widowed	81	0.043
**Education**		
None	87	0.001
Primary	61	0.041
Secondary	21	
Graduate	1	
Higher	3	
Occupation		
Self-employed	55	0.116
Employee	73	0.074
Unemployed	45	0.310

Data are number (n) or mean (standard error).

**Table-II:** Risk factors for HIV

*Factors*	*Number (n)*	*p value*
Blood donor	27	0.001
Injection drug user	135	
Blood transfusion recipients	11	
Sexual Behaviours		
Heterosexual	168	0.039
Homosexual	4	
Bisexual	1	
History of Sexually Transmitted Diseases		
Yes	39	0.341
No	41	0.420
Don’t know	93	0.115

Data are number (n).

It was observed that 87.28% HIV positive patients were urban dwellers (p = .035).The burden of HIV in the divorced/widowed (46.82%, p = .043) was more as compared to the unmarried (16.76%, p > .065) and married (36.41%, p > .178). Almost half (50.28%) of the infected subjects were illiterate and 35% had primary education.

HIV was significantly (p = .001) higher in uneducated and those having primary education (p = .041).Employment category was insignificant in diseased individuals, as 31.79% were self-employed (p = 0.116), 42.19% were employees (p = 0.074). Percentage of unemployed persons was 26% (p = 0.310).

A large proportion (78%) of the participants was injection drug users (IDU). Whereas, minor fractions (15.6% and 6.35%) were involved in unprotected blood donation and transfusion respectively. In statistical analysis, IDU was a constant causative factor (p = .001) for HIV infection. Blood donation and transfusion were not sensitive markers for the risk of HIV infections.

Multiple partners and extramarital relationships were apparently uncommon among local population. About 97% were heterosexual (P = 0.039) and were reluctant in disclosing the details of partners, if any. Self-reported history of sexually transmitted diseases (STDs) was 22.54% (p = 0.341). However, 53.75% (p = 0.115) had no knowledge of former STDs occurrence.

## DISCUSSION

The explosive spread of the human immunodeficiency virus (HIV) is a global health challenge that has both social and economic implications. Since 1987, when the first case of HIV was identified, slow but steady HIV incidence is documented in Pakistan. Given the lack of initial prevalence magnitude, and causative factors, indigenous HIV positivity and subsequently AIDS cases will rise. It is important to measure HIV prevalence, associated factors and institute preventive measures at early stages.^4^

Current investigation on HIV being the first from Faisalabad, Pakistan, quantified 0.54% HIV-1 and 0.009% HIV-2 prevalence. National Aids Control Programme (NACP) reported 0.052% and 0.064% HIV prevalence data for the years 1992 and 1995 respectively.^8^

Later on, 0.1% HIV prevalence was estimated by NACP in 2001.^9^ For the year 2007, 0.00542% HIV magnitude was stated.^10^ Most important finding of the study was higher HIV prevalence than the previous assessments. This reflects alarming situation that needs preventive interventions. Several regional studies from Lahore, Karachi, Peshawar and Northern areas are available. Khanani et al.^11^ and Mujeeb & Hashmi^12^ observed 0.73% and 0.147% HIV seroprevalence among general populations in Karachi. Reports from Lahore, Peshawar and Northern Pakistan cited 0.06%, 0.1% and 0.05% prevalence rates respectively.^13^^-^^15^

As regards age and gender distribution, our results were partially in compliance with the other studies, as an age range of 20-50 years, with median age of 35 is established for infection.^4 ^Present study identified 6:1 gender wise ratio (male:female) in HIV subjects. Previously, Male to female ratio of 5:1 (Kayani et al)^16^ and 2:1 (Iqbal and Rehan)^15^ were described.

Residential area, marital status, education and occupation are socio-economic determinants of HIV serostatus. Urban residence was positively significantly related to HIV status.^4^^,^^17^ Most of the HIV diagnosed subjects were urban residents. So, it can be concluded that rural populations are vulnerable to HIV to lesser extent. In addition, HIV prevention strategies should be more focused in urban Faisalabad. Contrary to current finding, Solomon et al.^18^ identified negligible difference in HIV prevalence in urban and rural Indian population.

Review of literature revealed that no research in Pakistan has examined the relation between the risk of HIV infection and marital status. In present research, married people had lower HIV infection. The matrimonial role in hindering HIV spread needs to be highlighted, as indicated by Shisana et al^19^, married people have less HIV occurrence than unmarried.

The proportion of individuals with infection was found to be inversely related to the literacy levels in the present study. The prevalence decreased as the educational level increased. Low literacy rate is key factor for HIV in the developing countries.^20^ It was determined by Kirunga and Ntozi^17^ that level of education affects HIV status. Religious and socio-economical contexts hinder new educational syllabi, especially those concerned with sex education in Pakistan.^21^

All the infected subjects in recent investigation were almost equally disseminated in three occupational categories. Hence, the livelihoods opted by subjects was not significant in acquisition of HIV. Similar to our inferences, Kirunga and Ntozi^17^ described insignificance of occupation in HIV development.

Four potential risk factors *viz.*blood donation/transfusion, Injecting drug use, sexual conducts (heterosexual, homosexual, bisexual) and history of STD were analysed by MANOVA. Drug use via injections was the main source of HIV transmission. Current nationwide estimations of HIV are based on large screening programs on high and low risk populations. Such information cannot portray accurate picture of IDU in the general population. The data indicated excessive HIV prevalence among IDU, suggesting an urgent need to educate public about hazardous reuse of syringes.^21^^,^^22^

Blood donation and transfusion activities were not potential risks for HIV in our study. Although a low prevalence of HIV is cited for Pakistan, the concerns of HIV epidemic in future are due to specific risky behaviours prevailing in Pakistani society, especially habitual blood donation and transfusion practices. Poor facilities and negligence in blood screening at public and private health care centers are common.^21^^-^^23^

STD may contribute to the spread of HIV. History of STD in study subjects was not a significant aspect for HIV. According to WHO, 0.3% HIV prevalence is present among Pakistani STD patients.^22^^,^^24^ Neither sexual preference nor absence of past STD had significant role in developing HIV. These inferences should be accepted with caution, as religious and social values not only minimize such tendencies but also impede the disclosure.

***Limitations of the study: ***This is the first study of HIV prevalence in Faisalabad and it provides insight on certain factors that may be contributing to the HIV epidemic. However, it had several limitations. First, high-risk groups (sex workers) were not included. Secondly, the study sample may be biased in that only those who visited STI clinic were involved. Thirdly, drug abuse and sexual behaviours are sensitive issues. The odds of their miscommunication must not be ignored.

## CONCLUSION

HIV is widely prevalent in Faisalabad region, Pakistan and this study provides an opportunity to restrict its further spread. High risk practices such as injection drug abuse, blood donation/transfusion needs to be amended and extramarital sexual contacts should be avoided.

## Authors Contributions:

**MAM: **Conceived and designed the study, data collection, **FH: **Conceived and designed the study, did manuscript writing, editing and review of final manuscript, **MJ**: Did biological testing, data collection.

## References

[1] Yousaf MZ, Zia S, Babar ME, Ashfaq UA (2011). The epidemic of HIV/AIDS in developing countries; the current scenario in Pakistan. Virol J.

[2] Singh S, Ambrosio M, Semini I, Tawil O, Saleem M, Imran M (2013). Revitalizing the HIV response in Pakistan: A systematic review and policy implications. Int J Drug Policy.

[3] Altaf A, Zahidie A, Agha A (2012). Comparing risk factors of HIV among hijra sex workers in Larkana and other cities of Pakistan: an analytical cross sectional study. BMC Public Health.

[4] Hyder AA, Omar AK (1998). HIV/AIDS in Pakistan: the context and magnitude of an emerging threat. J Epidemiol Community Health.

[5] Rajabali A, Khan S, Warraich HJ, Khanani MR, Ali SH (2008). HIV and homosexuality in Pakistan. Lancet Infect Dis.

[6] Khan AA, Khan A (2011). Performance and coverage of HIV interventions for injection drug users: Insights from triangulation of programme, field and surveillance data from Pakistan. Int J Drug Policy.

[7] Farid-ul-Hasnain S, Johansson E, Krantz G (2009). What do young adults know about the HIV/AIDS epidemic? Findings from a population based study in Karachi, Pakistan. BMC Infect Dis.

[8] (1995). National AIDS Control Program, HIV/AIDS Surveillance Project.

[9] (2005). National AIDS Control Program, HIV/AIDS Surveillance Project.

[10] (2008). USID HIV/AIDS Health Profile Pakistan. Surveillance Repor.

[11] Khanani RM, Hafeez A, Rab SM, Rasheed S (1987). AIDS and HIV associated disorders in Karachi. J Pak Med Assoc.

[12] Mujeeb SA, Hashmi MRA (1988). A study of HIV-antibody sera of blood donors and people at risk. J Pak Med Assoc.

[13] Raziq F, Alam N, Ali I (1993). Serosurveillance of HIV infection. Pak J Pathol.

[14] Tariq W, Malik IA, Hassan ZU (1993). Epidemiology of HIV infection in northern Pakistan. Pak J Pathol.

[15] Iqbal J, Rehan N (1996). Seroprevalence of HIV: six years’ experience at Sheikh Zayed Hospital, Lahore. J Pak Med Assoc.

[16] Kayani N, Sheikh A, Khan A, Mithani C, Khurshid M (1994). A view of HIV-1 infection in Karachi. J Pak Med Assoc.

[17] Kirunga CT, Ntozi JP (1997). Socio-economic determinants of HIV serostatus: a study of Rakai District, Uganda. Health Transit Rev.

[18] Solomon S, Kumarasamy N, Ganesh AK, Amalraj RE (1998). Prevalence and risk factors of HIV-1 and HIV-2 infection in urban and rural areas in Tamil Nadu, India. Int J STD AIDS.

[19] Shisana O, Zungu-Dirwayi N, Toefy Y, Simbayi LC, Malik S, Zuma K (2004). Marital status and risk of HIV infection in South Africa. S Afr Med J.

[20] Cleland J, Ferry B (1995). Sexual Behavior and AIDS in the developing world.

[21] Ali S, Khanani R, Tariq WU, Shah SA (1995). Understanding the context of HIV/AIDS infection in Pakistan. Venereol.

[22] Damani NN (1987). HIV antibodies testing in Pakistan. J Pak Med Assoc.

[23] Mujeeb SA (1993). Blood transfusion – a potential source of HIV/AIDS spread. J Pak Med Assoc.

[24] Shrestha PN (1996). HIV/AIDS surveillance in the Eastern Mediterranean Region. East Mediterr Health J.

